# Sodium Alginate Hydrogel with Zinc Ion Nanoparticles for Synergistic Neuroprotection and Functional Recovery in Spinal Cord Injury

**DOI:** 10.3390/md24050176

**Published:** 2026-05-13

**Authors:** Chuanxi Chi, Tianshun Ding, Xinping Han, Zongyu Wang, Qilong Cao, Liang Liu, Liming Li

**Affiliations:** 1 School of Rehabilitation Sciences and Engineering, University of Health and Rehabilitation Sciences, Qingdao 266113, China; chichuanxi@163.com (C.C.); dtianshun2022@163.com (T.D.); hanxp1003@163.com (X.H.); wzy2020bzmu@163.com (Z.W.); 2 College of Chemical and Biological Engineering, Shandong University of Science and Technology, Qingdao 266590, China; 3 Institute of Neurobiology, Binzhou Medical University, Yantai 264003, China; 4 Zhejiang Key Laboratory of Multiomics and Molecular Enzymology, Yangtze Delta Region Institute of Tsinghua University, Jiaxing 314006, China; 5 Department of Orthopedics, Beijing Luhe Hospital, Capital Medical University, Beijing 101149, China

**Keywords:** injectable hydrogel, zinc ion, spinal cord injury, neural regeneration, antioxidant

## Abstract

The current lack of effective treatments for traumatic spinal cord injury (SCI) presents a significant challenge in managing the complex microenvironmental alterations that follow the initial trauma. This study developed an injectable alginate hydrogel dynamically cross-linked by tannic acid–zinc nanoparticles (TA@Zn NPs), which exerts neuroprotective effects through the sustained release of zinc ions (Zn^2+^) and antioxidant TA@Zn NPs. TA@Zn NPs were cross-linked with phenylboronic acid-modified sodium alginate (SA) to form an injectable gel system. In response to the acidic and ROS-rich microenvironment characteristic of SCI, the hydrogel undergoes degradation, thereby triggering the disintegration of TA@Zn NPs and the concomitant release of Zn^2+^, enabling sustained therapeutic delivery. In a rat model of contusion injury, the degradation of TA@Zn NPs and the sustained release of Zn^2+^ significantly reduced oxidative damage and promoted axonal regeneration, which in turn inhibited scar formation and enhanced the tissue’s antioxidant capacity. Consequently, the group treated with the Zn^2+^-releasing hydrogel exhibited significant recovery of motor function. Collectively, these results validate the dual-function integration of Zn^2+^ as a dynamic cross-linker and neuroprotective agent within injectable hydrogels as a robust strategy for SCI repair, presenting a clinically translatable paradigm for neural regeneration.

## 1. Introduction

Spinal cord injury (SCI) constitutes a devastating trauma to the central nervous system, resulting in permanent loss of sensory, motor, and autonomic function below the injury level and imposing a substantial burden on patients, families, and society [1,2]. Following the primary mechanical insult, a complex cascade of secondary pathological events is initiated, encompassing oxidative stress, exacerbated inflammation, excitotoxicity, and glial scar formation, which collectively establish a hostile microenvironment detrimental to neural regeneration [3,4,5]. Currently, there is still a lack of clinical treatments for SCI that can effectively reverse nerve damage and promote functional recovery; the core challenge lies in the difficulty of precisely regulating the pathological microenvironment at the site of injury to support nerve repair [6,7,8]. Functional biomaterials, particularly hydrogels, have recently emerged as promising SCI therapeutic platforms by virtue of their excellent biocompatibility, injectability, and structural versatility [9,10]. In particular, injectable hydrogels have emerged as highly promising delivery platforms, enabling minimally invasive filling of irregular cavities, providing three-dimensional physical scaffolds, and serving as controlled-release vehicles for bioactive agents [11]. An optimal hydrogel scaffold requires favorable biocompatibility, appropriate mechanical properties, and intelligent responsiveness to pathological cues (including acidic pH and elevated ROS) for on-demand therapeutic release [12]. Among the numerous candidate neuroprotective agents, zinc ions are garnering increasing attention. Zinc is an essential trace element in humans, playing a pivotal role in modulating the activity of diverse enzymes and signaling pathways. Studies have demonstrated that local zinc ion concentrations undergo significant perturbation following SCI, with this homeostatic imbalance closely associated with neuronal death, oxidative damage, and exacerbated inflammation [13,14,15]. Exogenous zinc ions exert multifaceted neuroprotective effects by activating antioxidant pathways, modulating immune cell polarization, and suppressing scar formation, thereby promoting axonal regeneration [16,17,18]. However, direct delivery of free zinc ions is constrained by rapid systemic clearance, potential neurotoxicity, and the challenge of maintaining therapeutic concentrations at the lesion site.

To address these challenges, this study aims to develop an environmentally responsive injectable hydrogel system that integrates Zn^2+^ delivery with scaffold functionality. Sodium alginate (SA), as a natural polysaccharide, exhibits excellent biocompatibility, providing a hydrated microenvironment conducive to neural tissue integration and supporting cellular adhesion [19,20]. Tannic acid (TA) possesses multiple biological functions, including antioxidant, anti-inflammatory, and metal ion chelation activities, which collectively contribute to the attenuation of oxidative stress and inflammatory responses following SCI [21,22,23]. However, sodium alginate solution alone cannot form a stable gel scaffold at the lesion site, precluding the maintenance of effective local concentrations. Similarly, the administration of free TA or uncomplexed Zn^2+^ is constrained by rapid diffusion, accelerated systemic clearance, and potential neurotoxicity, thereby precluding sustained and controllable local therapeutic delivery. To address these limitations, this study employed a rational design strategy wherein TA and Zn^2+^ were pre-assembled into nanoparticles (TA@Zn NPs), which were subsequently dynamically cross-linked with phenylboronic acid-modified sodium alginate to construct a composite gel system integrating both scaffold functionality and controlled drug delivery capabilities. This system assembles into an injectable three-dimensional network via dynamic coordination between TA@Zn NPs and phenylboronic acid-modified sodium alginate. Upon injection into the ROS-rich, acidic SCI lesion milieu, the hydrogel undergoes stimuli-responsive degradation, triggering gradual disintegration of the nanoparticles and sustained Zn^2+^ release, thereby enabling prolonged modulation of the injured microenvironment. The sustained-release properties and biocompatibility of this hydrogel were validated in a rat contusion SCI model. Furthermore, the system demonstrated comprehensive therapeutic efficacy by mitigating oxidative damage, promoting axonal regeneration, inhibiting scar formation, and ultimately improving motor function recovery. This study establishes a robust strategy and provides experimental validation for the development of zinc-incorporated functional hydrogels as therapeutic platforms for spinal cord injury repair.

## 2. Results

### 2.1. Synthesis and Characterization of TA@Zn Nanoparticles

**Figure 1:**
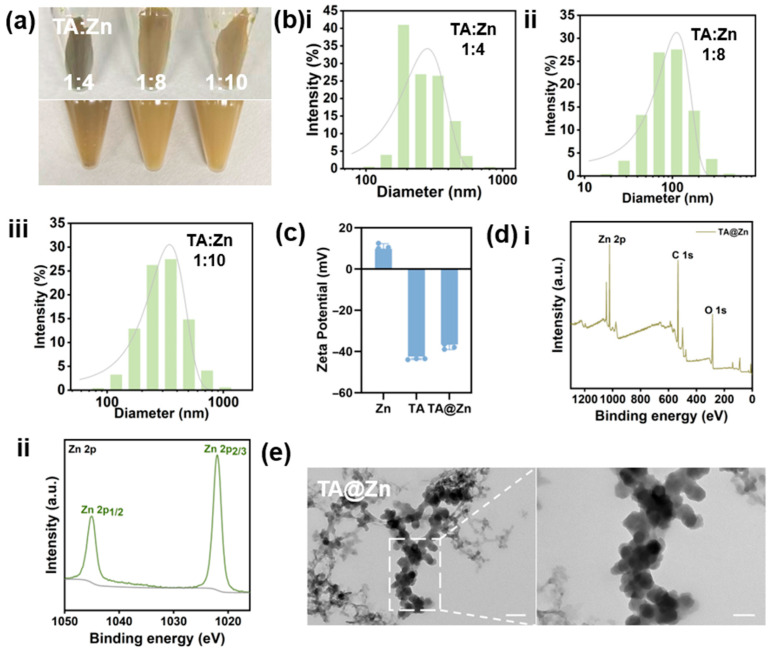
(**a**) Morphological images of nanoparticles with different molar ratios. (**b**) DLS image of TA@Zn nanoparticles with different molar ratios. (**c**) Zeta potential of TA@Zn, TA, and Zn. (**d**) XPS survey spectrum of TA@Zn nanoparticles. (**e**) TEM images of TA@Zn nanoparticles. Scale bars: 100 nm (unzoomed image in (**e**)), 50 nm (zoomed image in (**e**)).

To prevent burst release of ions, TA@Zn nanoparticles were synthesized via a facile one-pot method, wherein Zn^2+^ was coordinated in a single step with tannic acid—rich in catechol and pyrogallol moieties—to yield self-assembled nanostructures. The phenolic hydroxyl moieties coordinated with Zn^2+^ to mediate nanoparticle assembly. Figure 1a presents macroscopic images of TA@Zn nanoparticles synthesized at varying TA/Zn molar ratios, which exhibited a characteristic pale green coloration. The highest nanoparticle yield was observed at a TA/Zn molar ratio of 1:8. The hydrodynamic diameter of TA@Zn nanoparticles was determined by dynamic light scattering (Figure 1b), revealing an approximate size of 100 nm at the optimal 1:8 molar ratio. Figure 1c shows that the zeta potential of TA@Zn shifted to −37.83 mV following the addition of TA. Similarly, the XPS survey spectrum in Figure 1d(i) reveals characteristic peaks for C, O, and Zn on the surface of the TA@Zn sample, confirming the elemental composition of the nanoparticles and indicating the successful formation of TA-Zn nanoparticles. Furthermore, the Zn 2p XPS spectrum shown in Figure 1d(ii) exhibits binding energies of 1021.8 eV for Zn 2p_3/2_ and 1044.9 eV for Zn 2p_1/2_, respectively, indicating the presence of Zn^2+^ in the sample [24]. The morphology of TA@Zn nanoparticles was examined by transmission electron microscopy (TEM), revealing uniformly spherical nanostructures with an average diameter of approximately 100 nm (Figure 1e). These findings confirmed the successful fabrication of TA@Zn nanoparticles.

### 2.2. Synthesis and Characterization of Hydrogels

**Figure 2:**
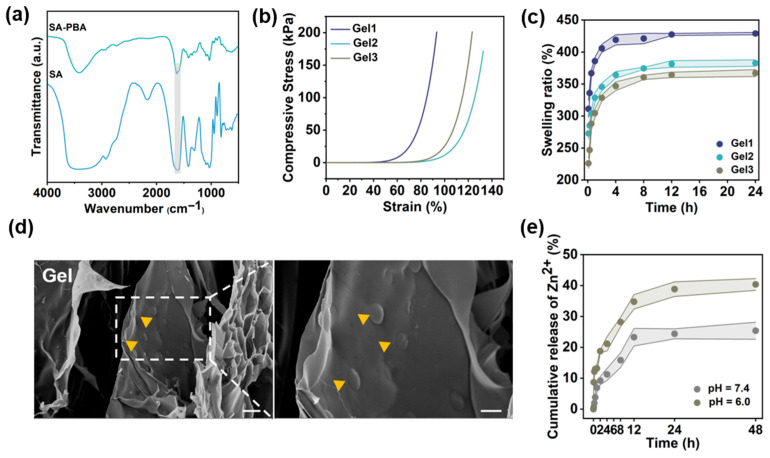
(**a**) FTIR spectra of SA and SA-PBA. (**b**) Compressive strain-stress of gel hydrogels. (**c**) Swelling behavior of gel hydrogels during 24 h. (**d**) SEM image of the hydrogel. Scale bar: 40 μm (unzoomed image in (**d**)) and 20 μm (zoomed image in (**d**)). (**e**) The release of zinc ions from gel hydrogels under different conditions.

In order to achieve the sustained release of zinc ions and prevent sudden release, Zn^2+^ was incorporated into the hydrogel matrix in the form of TA@Zn NPs. The polyphenolic structures present on the surface of TA@Zn NPs function as cross-linking sites for sodium alginate modified with phenylboronic acid (SA-PBA), thereby facilitating hydrogel formation. As demonstrated in Figure 2a, the FTIR analysis of SA-PBA revealed an absorption peak corresponding to the aromatic C-H bending vibration at 770 cm^−1^, as well as a characteristic absorption peak of the benzene ring at 1591 cm^−1^. Furthermore, the presence of B-O stretching vibrations was indicative of the successful grafting of phenylboronic acid onto SA. The mechanical properties of the hydrogel were then subjected to further evaluation (Figure 2b). The outcomes of the compression test indicated that all hydrogels exhibited excellent compressive strength. Specifically, Gel1 and Gel3 had maximum compressive strengths of 201.06 kPa and 201.46 kPa, respectively, while Gel2 had a maximum compressive strength of 171.78 kPa. It is noteworthy that Gel2 demonstrated exceptional resistance to deformation during compression, suggesting its high structural stability. The equilibrium swelling behavior of hydrogels with different compositions was examined over a 24 h period. As demonstrated in Figure 2c, Gel1, Gel2, and Gel3 exhibited swelling ratios of 428.89%, 382.75%, and 350.04%, respectively. These results indicate that the gels exhibit excellent water absorption capacity, suggesting that they can effectively absorb excess tissue fluid from the injured site. SEM images revealed that the TA@Zn nanoparticles were uniformly distributed across the surface of the hydrogel and exhibited a porous internal structure (Figure 2d). This interconnected network of pores facilitated the exchange of nutrients and gases [25,26]. Figure 2e demonstrates the cumulative release behavior of Zn^2+^ from the Gel2 hydrogel in a variety of release media. The results demonstrated that zinc ions were released rapidly within 12 h, reaching a steady state of release after 24 h. Notably, the cumulative release rate was accelerated under acidic conditions compared to physiological environments. This finding suggests that the acidic microenvironment promotes hydrogel network dissociation, thereby facilitating zinc ion release. In summary, the results indicate that the hydrogel possesses suitable swelling properties, a microporous structure, and robust mechanical properties. These characteristics enable it to effectively fill post-injury cavities, provide mechanical cushioning at the injury site, and mitigate secondary damage, thereby meeting the fundamental requirements for spinal cord injury repair.

### 2.3. Gel Hydrogel Promotes Functional Recovery and Ameliorates Lesion Volume After Spinal Cord Injury

**Figure 3:**
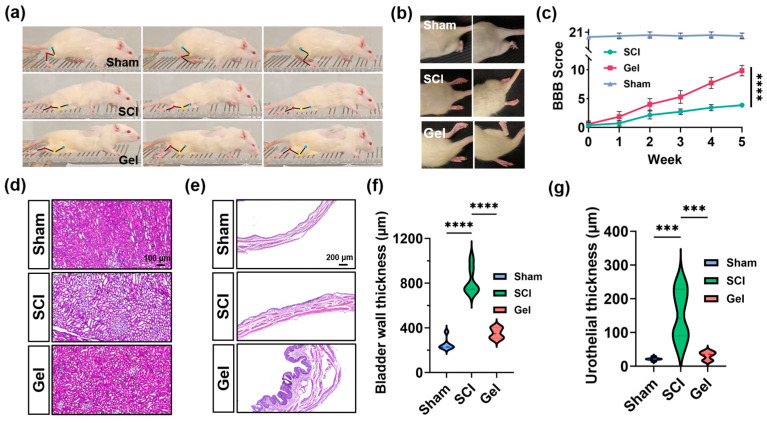
(**a**) Representative gait of rats in the ladder-climbing test on day 35. (**b**) Hindlimb activity of rats after 5 weeks. (**c**) BBB score recovery of hindlimb motor function at 5 weeks (each group of three or more separate samples). (**d**) HE staining results of major organs (kidneys). (**e**) HE staining results of bladder tissue. (**f**) Thickness of the bladder wall. (**g**) Thickness of the urothelium. Data are shown as mean ± SEM, *** *p* < 0.001, **** *p* < 0.0001, *n* ≥ 3.

Motion snapshots revealed that rats in the Gel group exhibited improved spinal curvature and a more even distribution of limb forces during ladder climbing, which may contribute to enhanced postural stability (Figure 3a). Consistently, relative to the SCI group, the Gel group showed a significantly higher proportion of effective gaits (Figure 3b). As assessed by BBB scoring (Figure 3c), motor recovery in the SCI group was markedly slower than that in the sham group. In contrast, gel treatment produced a time-dependent improvement in hindlimb motor function. Collectively, these findings suggest that the gel hydrogel promotes recovery of overall gait performance and fine motor coordination in rats with SCI.

Figure 3d presents HE staining of rat kidney sections. Compared with the sham group, the SCI group showed vacuolar degeneration of renal tubular epithelial cells, tubular dilation, and cast formation, indicating secondary renal injury following SCI. These pathological changes were attenuated in the Gel group, with partial restoration of tubular architecture and reduced vacuolar degeneration and cast formation. Thus, gel hydrogel treatment may partially mitigate the progression of SCI-associated renal injury, potentially driven by urinary retention and chronic inflammation, by improving the local microenvironment.

HE staining of bladder sections demonstrated pronounced pathological alterations in the SCI group, including marked bladder wall thickening and disorganized smooth muscle bundles (Figure 3e). In the Gel group, these changes were partially alleviated, as evidenced by reduced bladder wall thickening, a relatively more organized muscle fiber arrangement, and improved urothelial hyperplasia; however, mild tissue edema and irregular mucosal architecture persisted. These results indicate that hydrogel therapy exerts partial protective effects against bladder pathology following SCI, potentially attenuating aberrant thickening of the bladder wall and urothelium by ameliorating urinary retention and modulating the local microenvironment.

### 2.4. Gel Hydrogel Promotes Axonal Regeneration and Functional Recovery After Spinal Cord Injury

**Figure 4:**
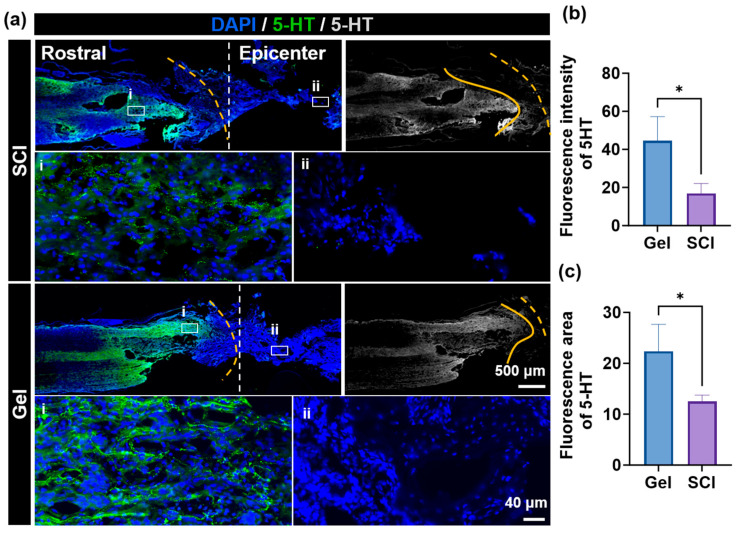
(**a**) Representative immunofluorescence images of spinal cord sections showing 5-HT (green) immunoreactivity at the lesion site for the SCI and Gel groups. (**b**) Fluorescence intensity of 5-HT. (**c**) Fluorescence area of 5-HT. Data are shown as mean ± SEM; * *p* < 0.05, *n* ≥ 3.

Immunofluorescence staining was used to examine 5-HT expression in spinal cord tissue to assess the effect of gel treatment on the descending serotonergic pathway (Figure 4). In the SCI group, the central region of the lesion exhibited a significant reduction in 5-HT-positive fibers with a disorganized arrangement. In the rostral (cephalic) region of the lesion, a small number of severed and retracted fibers were observed, accompanied by markedly attenuated fluorescent signals. These findings indicate that spinal cord injury disrupts the continuity of the descending serotonergic pathway. In contrast, the Gel group exhibited significantly improved 5-HT immunoreactivity. Compared with the SCI group, the pro-lesional region of the Gel group contained a greater number of morphologically intact 5-HT-positive fibers. Furthermore, fine regenerating fibers were observed extending from the pro-lesional region toward the lesion center, indicating active axonal regeneration. These results suggest that gel treatment promotes either the regeneration of damaged serotonergic axons or the preservation of their structural integrity, potentially contributing to the recovery of motor function following injury.

### 2.5. Hydrogels Reduce Scarring and Promote Nerve Regeneration After Spinal Cord Injury

**Figure 5:**
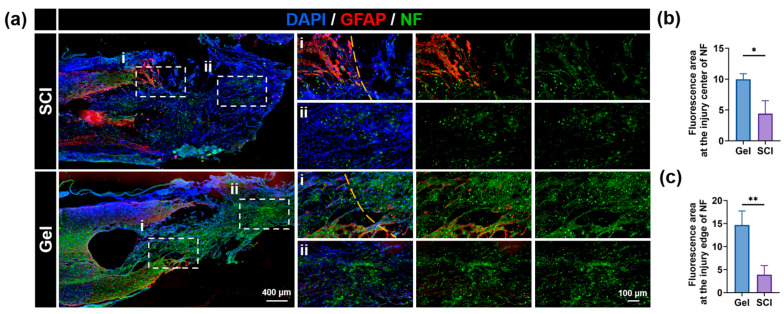
(**a**) Representative immunofluorescence images of spinal cord sections showing GFAP (red) and NF (green) immunoreactivity at the lesion site for the SCI and Gel groups. (**b**) Fluorescence area at the injury center of NF. (**c**) Fluorescence area at the injury edge of NF. Data are shown as mean ± SEM; * *p* < 0.05, ** *p* < 0.01, *n* ≥ 3.

Glial scar is mainly composed of high expression of glial fibers acidic protein (GFAP) of reactive astrocytes. These cells form a physical barrier, hindering nerve regeneration after spinal cord injury. This study investigated the effects of gel hydrogel implantation on glial scar formation at the injury site by GFAP immunofluorescence staining. The results showed that the number of GFAP-positive reactive astrocytes in the lesion area was significantly increased in the untreated SCI group (Figure 5a). Conversely, the gel hydrogel treatment group showed significantly reduced GFAP-positive cells (Figure 5a), indicating that the hydrogel effectively suppressed glial scar formation, thereby promoting SCI repair. Axon regeneration in the damaged region was then evaluated. Neurofilament (NF) staining showed that the SCI group exhibited severe axonal damage, characterized by obvious atrophy. In contrast, the hydrogel treatment group showed a large number of NF-positive filaments extending to the injury center and adjacent areas. Quantitative analysis further confirmed that gel hydrogel treatment significantly increased NF expression levels in the central and peripheral lesion areas compared to the SCI group (Figure 5b,c). The enhanced axonal regeneration suggested that zinc ions released from the hydrogel could restore local zinc levels, thus facilitating nerve regeneration. In summary, the gel hydrogel promotes functional recovery after spinal cord injury by alleviating local zinc deficiency and inhibiting glial scar formation, thereby creating a favorable microenvironment for nerve regeneration.

### 2.6. Gel-NP Hydrogel Mitigates Oxidative Damage and Modulates Immune Microenvironment to Promote Spinal Cord Injury Repair

**Figure 6:**
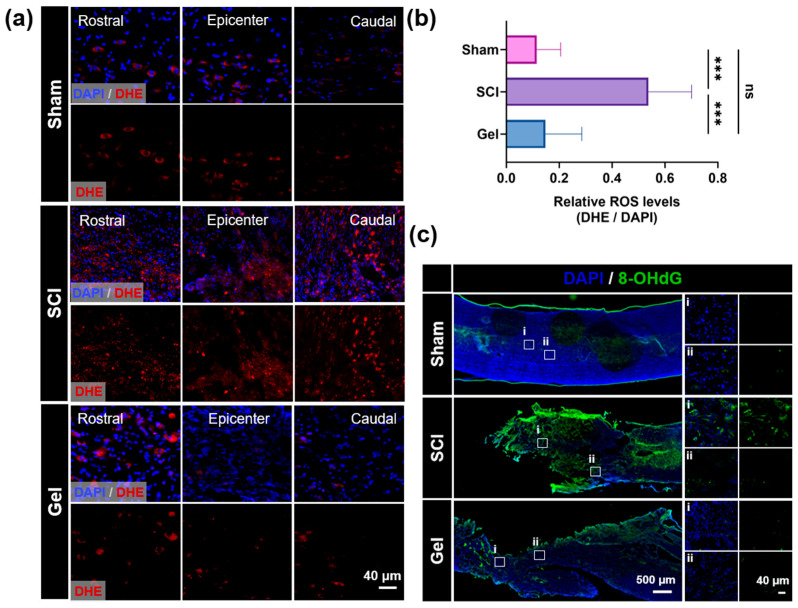
(**a**) Representative immunofluorescence images of spinal cord sections showing DHE (red) immunoreactivity for the sham, SCI, and Gel groups. (**b**) Quantitative analysis of relative reactive oxygen levels, expressed as the ratio of DHE and DAPI fluorescence intensities. (**c**) Representative immunofluorescence images of spinal cord sections showing 8-OHdG (green) immunoreactivity for the sham, SCI, and Gel groups. Data are shown as mean ± SEM; *** *p* < 0.001, ns (not significant), *n* ≥ 3.

In the cascade of secondary damage following spinal cord injury (SCI), reactive oxygen species (ROS)-induced oxidative stress represents a critical factor that drives neuronal apoptosis and impedes axonal regeneration. Dihydroethidium (DHE) fluorescence staining was employed to detect and localize superoxide anions. Figure 6a shows only faint DHE background fluorescence in the spinal cord tissue of the sham group, indicating that ROS levels are maintained at extremely low levels under physiological conditions. In the SCI group, the area surrounding the injury exhibited dense and intense red fluorescence signals. Quantitative analysis revealed that the average fluorescence intensity was significantly higher than that in the sham group, demonstrating that severe oxidative stress occurred following SCI. In the gel hydrogel treatment group, DHE fluorescence intensity at the injury site was significantly lower than that in the SCI group, with a reduction of approximately 8.64% (Figure 6b). This indicates that the gel hydrogel effectively captures and scavenges excess superoxide anions at the injury site, thereby restoring redox homeostasis.

To further assess oxidative stress-induced cellular damage, particularly at the DNA level, immunofluorescence staining for 8-hydroxy-2′-deoxyguanosine (8-OHdG), a specific marker of oxidative DNA damage, was performed. As shown in Figure 6c, virtually no 8-OHdG-positive cells were detected in the sham group. By contrast, the SCI group exhibited intense 8-OHdG-positive fluorescent signals, indicating that oxidative stress induced DNA damage. However, the number of 8-OHdG-positive cells was significantly reduced in the Gel-NP hydrogel treatment group, indicating that Gel-NPs effectively protected cellular genetic material by alleviating oxidative stress, thereby attenuating oxidative stress-induced apoptosis.

### 2.7. Gel-NP Hydrogel Reprograms Macrophage Polarization to Facilitate Spinal Cord Injury Repair

**Figure 7:**
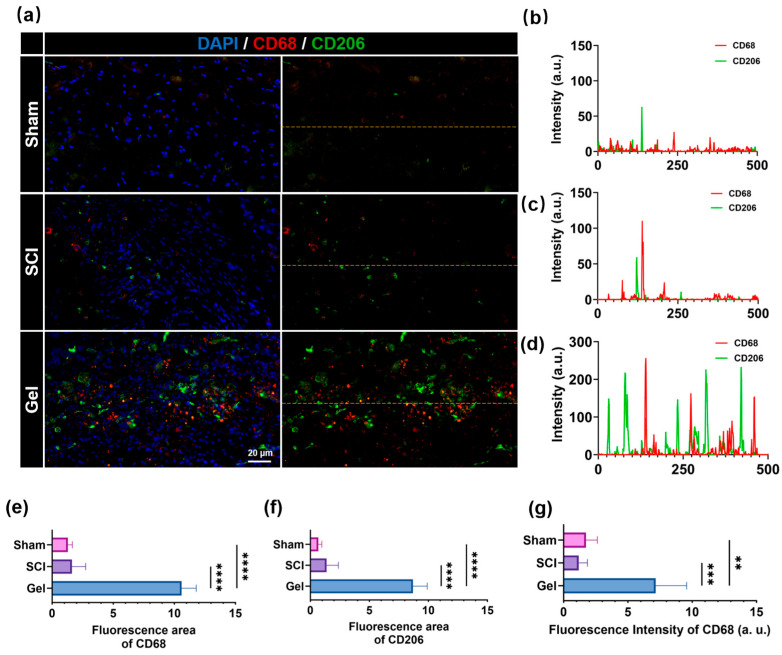
(**a**) Representative immunofluorescence images of spinal cord sections showing CD68 (red) and CD206 (green) immunoreactivity for the sham, SCI, and Gel groups. (**b**) Line-scan analysis of CD68 and CD206 fluorescence intensity profiles along the indicated distances in the sham group. (**c**) Line-scan analysis of CD68 and CD206 fluorescence intensity profiles along the indicated distances in the SCI group. (**d**) Line-scan analysis of CD68 and CD206 fluorescence intensity profiles along the indicated distances in the Gel group. (**e**) Fluorescence area of CD68. (**f**) Fluorescence area of CD206. (**g**) Fluorescence intensity of CD68. Data are shown as mean ± SEM; ** *p* < 0.01, *** *p* < 0.001, **** *p* < 0.0001, *n* ≥ 3.

Immunofluorescence staining revealed that CD68^+^ and CD206^+^ signals were barely detectable in the spinal cord tissue of the Sham group, indicating that macrophages/microglia remained in a quiescent state under physiological conditions (Figure 7). In the SCI group, sporadic CD68^+^ and CD206^+^ signals were observed, albeit with relatively low overall fluorescence intensity. By contrast, the gel-treated group exhibited markedly enhanced CD68^+^ and CD206^+^ signals at the lesion site, with evident spatial colocalization of both markers. Quantitative analysis demonstrated that the CD68-positive area, CD206-positive area, and CD68 fluorescence intensity in the Gel group were all significantly elevated compared to the SCI group (approximately 5- to 6-fold). These findings suggest that hydrogel implantation promotes the recruitment of macrophages/microglia to the injury site, concomitant with upregulated expression of the M2 phenotype marker CD206.

## 3. Discussion

In this study, we developed a composite platform by incorporating zinc–tannic acid nanoparticles into a phenylboronic acid-modified sodium alginate hydrogel. This hierarchical architecture helps minimize the burst release of metal ions and lowers the risk of local toxicity. Under the mildly acidic and ROS-enriched conditions characteristic of spinal cord injury, the hydrogel gradually degrades, allowing sustained release of Zn^2+^. Tannic acid, a natural polyphenolic compound, possesses abundant pyrogallol moieties that confer excellent free radical scavenging capacity and metal ion chelating properties, enabling it to exert synergistic antioxidant effects within the injured microenvironment. The sustained release of zinc ions helps eliminate excessive reactive oxygen species, alleviate oxidative stress, and synergistically modulate microglial activation, promoting their transition toward a tissue-repair-supportive phenotype, thereby facilitating the regulation of the local immune microenvironment. In addition, the system supports axonal regeneration. Through these coordinated antioxidant and immunomodulatory effects, the platform provides neuroprotective benefits and facilitates functional recovery after spinal cord injury.

### 3.1. Coordination-Based Material Design Enables Targeted Therapy

Zinc ions are essential regulators of numerous physiological activities [27,28,29]. Here, Zn^2+^ was coordinated with tannic acid at varying molar ratios to form nanoparticles, which were further integrated into a phenylboronic acid-modified sodium alginate hydrogel to establish a composite platform. Tannic acid, a natural polyphenol, possesses abundant pyrogallol moieties that not only confer excellent free radical scavenging capacity and metal ion chelating properties but also enable interactions with various biomacromolecules through hydrogen bonding and hydrophobic interactions, endowing it with multiple biological activities, including anti-inflammatory, antibacterial, and cell adhesion-promoting effects. Owing to the responsiveness of boronate ester bonds to the spinal cord injury microenvironment, the hydrogel network undergoes cleavage under pathological conditions, resulting in the gradual release of the nanoparticles. Concurrently, the coordination structure of the nanoparticles is progressively disrupted, permitting sustained Zn^2+^ release for localized therapeutic delivery. During this process, accompanied by the dissociation of coordination bonds, tannic acid molecules are also gradually released. Their polyphenolic structures can directly scavenge reactive oxygen species at the injury site and interact with endogenous metal ions, thereby further suppressing oxidative stress cascade reactions. Further studies on the swelling behavior of hydrogels have shown that PBA-modified sodium alginate hydrogels exhibit moderate water absorption. While maintaining the stability of their three-dimensional network, they are also capable of absorbing excess tissue fluid, which aids in the release of therapeutic factors within the damaged microenvironment.

### 3.2. Multimodal Evidence Demonstrates Functional Recovery After SCI

The therapeutic efficacy of hydrogels in the repair of spinal cord injury was evaluated using a multiparameter system [30,31]. Immunofluorescence staining revealed that the gel treatment group exhibited improved neuronal survival rates, enhanced axonal regeneration, and increased density of lower serotonergic fibers, while glial scar formation was reduced. The levels of oxidative stress markers were found to be significantly reduced, and microglia were observed to polarize towards the anti-inflammatory M2 phenotype. Behavioral analysis demonstrated that there was a sustained improvement in the BBB score, and no significant pathological changes were observed in the HE staining of the kidneys and bladder. These findings indicate that there is good in vivo biocompatibility.

In general, it is evident that the hydrogel under consideration effectively promotes functional recovery following spinal cord injury. This is attributable to the synergistic action of its antioxidant, anti-inflammatory, and neuroprotective properties.

### 3.3. Limitations and Future Directions

Although this study demonstrates the potential of the gel hydrogel for treating SCI models, several limitations should be acknowledged. First, this study utilized only in vivo rodent models, which exhibit significant pathologic differences from humans. It is therefore evident that overcoming interspecific differences will be necessary to achieve clinical translation. Second, spinal cord injury triggers a complex series of events, including neuronal apoptosis, blood vessel rupture, and glial scarring, and it is unlikely that a single material system will comprehensively address all these pathological processes. Third, functional recovery depends on both local microenvironment remodeling and the reconstruction of complex neural circuits. Despite the observed behavioral improvements, critical issues remain unresolved, particularly whether regenerated axons can establish functional synaptic connections. Fourth, the long-term safety and biodegradability of the material need to be validated through long-term follow-up studies. Fifth, as a proof-of-concept investigation of a novel biomaterial system, this study did not include a positive control group treated with a standard clinical drug such as methylprednisolone, a decision based on the bioinert nature of the alginate carrier. Moreover, substantial variations in animal strains, injury models, administration routes, and evaluation metrics across different studies limit the reliability of indirect comparisons with previously reported methylprednisolone data. Therefore, to fully establish the translational relevance of this strategy, future work should not only include long-term follow-up assessments of safety and material degradation but also perform direct, head-to-head comparisons with standard pharmacotherapy in the same animal model, which represents a key direction of our ongoing research.

## 4. Materials and Methods

### 4.1. Synthesis and Characterization of TA@Zn Nanoparticles

Synthesis of TA@Zn nanoparticles: TA@Zn nanoparticles were synthesized using a one-pot method. The brief steps are as follows: The following substances were dissolved in deionized water: 22.92 mg of ZnSO_4_·7H_2_O (Macklin Co., Ltd., Shanghai, China) and 17 mg of tannic acid (TA, purchased from Energy Chemical Co., Ltd., Shanghai, China). The mixture was stirred continuously at 400 rpm at room temperature using a digital magnetic stirrer for 24 h, with the pH maintained at 8.0. Subsequent to the completion of the reaction, the reaction mixture was transferred to a 15 mL centrifuge tube and subjected to centrifugation at 9000 rpm for a duration of 20 min. The superior portion of the mixture was then discarded, with the precipitate collected. The pellet was resuspended in deionized water, vortexed, and then subjected to a second round of centrifugation and washing under the same conditions. The process of purification continued until the absence of coloration in the resulting medium was apparent. This is indicative of the successful isolation and subsequent formation of TA@Zn NPs. Using the same procedure, TA@Zn NPs with molar ratios of 1:4 and 1:10 were synthesized. The hydrodynamic diameter and zeta potential were measured using a particle size analyzer (NanoBrook 90Plus Zeta, Bruker, New York, NY, USA). The TA@Zn NPs were dispersed in deionized water at a concentration of 0.1 mg/mL by ultrasonication at room temperature. The surface chemical composition and elemental states were analyzed by X-ray photoelectron spectroscopy (XPS, Thermo Fisher Scientific, Waltham, MA, USA) using a monochromatic Al Kα source (1486.6 eV). The chemical structure and functional groups were characterized by Fourier-transform infrared spectroscopy (FTIR, Bruker, Billerica, MA, USA) in the range of 4000–400 cm^−1^. The morphology and size distribution were observed by transmission electron microscopy (TEM, JEOL, Akishima, Japan) at an accelerating voltage of 200 kV.

Synthesis of SA-PBA: The sodium alginate (SA, 4 g, Macklin Co., Ltd., Shanghai, China) was dissolved in 400 mL of deionized water. Subsequently, 3.84 g of EDC and 3.45 g of NHS (Macklin Co., Ltd., Shanghai, China) were added to the aqueous solution. Subsequent to the complete dissolution, a quantity of 1.56 g of 3-aminophenylboronic acid (Acmec Biochemical Technology Co., Ltd., Shanghai, China) was added. The mixture was stirred at room temperature for 24 h, then dialyzed in ultrapure water for 3 days (retention molecular weight 3.5 kDa, purchased from Changsha Yibo Biotechnology Co., Ltd., Changsha, China), and finally freeze-dried to obtain SA-PBA. The chemical structure was characterized by ^1^H nuclear magnetic resonance (^1^H NMR, Bruker, Billerica, MA, USA) and Fourier-transform infrared spectroscopy (FTIR, Bruker, Billerica, MA, USA). ^1^H NMR spectra were recorded at 500 MHz using deuterated solvents. FTIR spectra were recorded in the ATR mode from 4000 to 400 cm^−1^.

Synthesis of SA-PBA/SA/TA@Zn (Gel) Hydrogel: In a suitable container, separate amounts of SA and SA-PBA were weighed out and dissolved in water (6% *w*/*v* and 10% *w*/*v*, respectively) to yield aqueous solutions. Equal volumes were mixed to obtain Solution A. In order to prepare Solution B, it is necessary to prepare two separate 2% (*w*/*v*) TA@Zn solutions and two 2.5% (*w*/*v*) Zn^2+^ solutions. These should then be mixed in equal volumes to obtain Solution B. Subsequently, Solutions A and B were amalgamated at volume proportions of 2:1, 1:1, and 1:2 and vigorously agitated using a vortex mixer. In order to induce gelation, it was necessary to adjust the pH level to the range of 7.2–7.4. The three-dimensional porous architecture and surface morphology were visualized by scanning electron microscopy (SEM, ZEISS AG, Oberkochen, Germany) at an accelerating voltage of 5 kV. Prior to imaging, the samples were sputter-coated with a thin layer of gold (approximately 10 nm) to enhance conductivity.

### 4.2. Testing Methods—In Vitro

#### 4.2.1. Swelling Ratio of Gels

To investigate the swelling behavior of the hydrogel, freeze-dried gel samples (3 mm × 8 mm) were immersed in phosphate-buffered saline (PBS, pH 7.4) at 37 °C until swelling equilibrium was attained. The swelling ratio in the dry state (*Q*) was subsequently calculated using the following formula:

$$Q(\%)=\frac{M_{t}-M_{0}}{M_{0}}\times 100$$

where *M_t_* is the mass of the hydrogel measured at various time intervals after surface moisture has been removed by wiping, and *M*_0_ is the initial mass of the hydrogel following freeze-drying.

#### 4.2.2. Characterization of the Mechanical Properties of Gels

To investigate the mechanical properties of the gel hydrogel, cylindrical specimens (3 mm in diameter × 8 mm in height) were subjected to compression testing using a MARK-10 universal testing machine (Mark-10 Corporation, Copiague, NY, USA) at a crosshead speed of 10 mm/min to evaluate their stress–strain behavior.

#### 4.2.3. Cumulative Release Behavior of Zinc Ions

In accordance with the manufacturer’s protocol for the zinc ion colorimetric assay kit (Biyuntian, Shanghai, China), zinc ion release was quantified at predetermined time intervals. The cumulative release of zinc ions was quantified using a commercial zinc ion detection kit. Release studies were performed using 10 mL of phosphate-buffered saline (PBS, pH 7.4) and 10 mL of PBS (pH 6.0) as the release media. The hydrogel samples were immersed in the respective media and incubated at 37 °C with constant shaking at 100 rpm. At predetermined time points, 1 mL of the release medium was withdrawn for analysis and replaced with an equal volume of fresh medium to maintain sink conditions.

### 4.3. Testing Methods—In Vivo

#### 4.3.1. General Design

Female Sprague Dawley (SD) rats (220–250 g) were obtained from Beijing HFK Bioscience Co., Ltd., Beijing, China. for SCI model establishment. The animals were housed under controlled environmental conditions (temperature 24 ± 1 °C, relative humidity 55 ± 5%, 12 h light/dark cycle) and had free access to standard feed and sterile water. Rats were anesthetized with 3% sodium pentobarbital administered via intraperitoneal injection. After the induction of anesthesia, an incision was made at the level of the T8 vertebra, with a midline orientation. The T7–T8 laminae are then exposed by blunt dissection, after which the attached muscles are resected in order to facilitate laminectomy. In the following step of the experiment, the spinal segments beneath underwent exposure, following which a contusion injury was inflicted. The rats were randomly divided into three groups (*n* = 6 per group): the sham-surgery group, the spinal cord injury group, and the spinal cord injury plus gel group. Immediately after the spinal cord contusion model was established, 40 μL of hydrogel was injected into the injury site. Following the surgical procedure, the muscle layer and the skin layer were sutured in sequence. In order to prevent infection, it is necessary to administer penicillin intraperitoneally for a period of seven consecutive days. The bladder must be manually expressed on two occasions each day until the patient is able to urinate spontaneously.

#### 4.3.2. Assessment of Motor Function

The 21-point Basso–Beattie–Bresnahan (BBB) Motor Function Scale was utilized to evaluate motor function in rats. This scale evaluates both general motor ability and fine foot movements over an extended observation period. The hindlimb function of the rats was evaluated using a horizontal staircase test. The measurement process commenced in the first week following the surgical procedure, with BBB scores being recorded on a weekly basis until the fifth week. Prior to the commencement of the test, the rats underwent a three-day standard acclimatization period during the fifth week. Subsequently, the steps were adjusted to an irregular spacing pattern in order to accurately assess proprioception and limb coordination.

#### 4.3.3. Tissue Processing

Subsequently, rats were deeply anesthetized and subjected to cardiac perfusion with a solution of isotonic saline, followed by 4% paraformaldehyde (AR1068). Utilize the center of the injury as a reference point and excise a 1.5 cm segment of spinal cord tissue. The tissue should then be placed in 4% paraformaldehyde and fixed for 24 h at 4 °C. Following this, the tissue should be transferred to a 30% sucrose solution for cryoprotection until the tissue has completely settled to the bottom and reached a state of saturation.

#### 4.3.4. HE Staining

The collection of organ and tissue samples from rats was conducted, followed by an overnight fixation process at a temperature of 4 °C, using 4% paraformaldehyde (BL539A, Bioshark, Shanghai, China). Following rectification, the samples should be transferred to a solution of 50% ethanol and stored at a temperature of 4 °C. The samples were then dehydrated using an ethanol gradient, embedded in paraffin wax, and sectioned. Following staining of the sections with hematoxylin (BL735B, Bioshark, Shanghai, China), a process of differentiation and re-staining was initiated. Thereafter, counterstaining with eosin (Bioshark, Shanghai, China, BL735B) was conducted. Subsequent to a procedure of dehydration and clarification with xylene, neutral gum (G8590, Solarbio, Shanghai, China) was used for mounting the slides. Frozen spinal cord sections (5 µm) were processed using a procedure similar to the previously described method.

#### 4.3.5. Immunohistochemistry

The spinal cord tissue underwent sectioning following fixation, dehydration, and embedding in OCT. The sections were then subjected to a thorough wash with phosphate-buffered saline (PBS), followed by antigen retrieval. To ensure optimal blocking, the sections were then immersed in a blocking solution composed of PBS with 0.2% Triton X-100 and 10% donkey serum. The primary antibody was then subjected to an overnight incubation at a temperature of 4 °C. Following a thorough wash with phosphate-buffered saline (PBS), the fluorescent secondary antibody (donkey anti-rabbit IgG, conjugated with Alexa Fluor 488 or 594, Proteintech, Wuhan, China) was added, and the mixture was incubated at 37 °C for a period of 40 min. Following a subsequent wash, the slides were mounted using a fluorescent mounting medium (Bioshark, Shanghai, China) and imaged using a 3DHISTECH slide scanner, and the fluorescence intensity was quantified using ImageJ 1.8.0 software.

#### 4.3.6. Statistical Analysis

All data were analyzed using GraphPad Prism 10.1.2 and are presented as mean ± standard deviation (SD). Comparisons among multiple groups were performed using one-way analysis of variance (ANOVA) followed by Tukey’s post hoc test. Statistical significance was set at *p* < 0.05. All experiments were performed with a minimum of three biological replicates unless otherwise stated.

## 5. Conclusions

In summary, the microenvironment-responsive hydrogel platform developed in this study is capable of sustaining the release of Zn^2+^, thereby exerting a synergistic effect in terms of antioxidant and anti-inflammatory properties and promoting neuroprotection and functional recovery following spinal cord injury. By responding to pathological cues and enabling controlled release, it provides a solid foundation for use in complex injury scenarios. Subsequent studies will entail the assessment of its effectiveness in primate models and the additional elucidation of the molecular mechanisms through which this platform regulates the immune microenvironment. This will serve to enhance its potential for clinical translation.

## Data Availability

The data presented in this study are available upon request from the corresponding authors.
